# Newborn Body Perception: Sensitivity to Spatial Congruency

**DOI:** 10.1111/infa.12083

**Published:** 2015-04-22

**Authors:** Maria Laura Filippetti, Giulia Orioli, Mark H Johnson, Teresa Farroni

**Affiliations:** Department of PsychologyRoyal Holloway University of London; Dipartimento di Psicologia dello Sviluppo e della SocializzazioneUniversità degli Studi di Padova; Centre for Brain and Cognitive Development, Birkbeck CollegeUniversity of London

## Abstract

Studies on adults have demonstrated that the perception our own body can be manipulated by varying both temporal and spatial properties of multisensory information. While human newborns are capable of detecting the temporal synchrony of visuo-tactile body-related cues, it remains unknown whether they also utilise spatial information for body perception. Twenty newborns were presented with a video of an infant’s face touched with a paintbrush, while their own face was touched either in the spatially congruent, or an incongruent, location. We found that newborns show a visual preference for spatially congruent synchronous events, supporting the view that newborns have a rudimentary sense of their own body.

Multisensory integration is critical for human social interaction and for our perception of our own bodies (Bahrick & Lickliter, 2002; Gibson, 1979; Longo & Haggard, 2012). The efficient integration of information between the sense modalities through the detection of temporal and spatial contingency not only allows individuals to make sense of the various sensory inputs coming from the environment, but also potentially defines the self as differentiated from others (Bahrick & Lickliter, 2002, 2012). Adult studies that have investigated body awareness have based their research on the use of perceptual illusions, whereby through the integration and disruption of multisensory inputs (usually visual and tactile cues) ownership and location over body-parts and full body is manipulated (Botvinick & Cohen, 1998; Lenggenhager, Tadi, Metzinger, & Blanke, 2007; Petkova & Ehrsson, 2008; Tsakiris, 2008). These studies provided relevant insight into perceptual components involved in the construction and updating of body representation.

Crucially, intermodal perception is well accomplished in humans, and even newborns are able to process such information in a coherent way (Lewkowicz & Ghazanfar, 2006, 2009; Lewkowicz, Leo, & Simion, 2010). Evidence such as this suggests that integration of sense modalities may be present from birth (or earlier) helping human newborns to overcome the “blooming, buzzing confusion” (James, 1890, p. 462). However, the question of how newborns’ intermodal perception informs their sense of bodily self has remained less well understood.

Recently, we demonstrated that human newborns are able to discriminate the temporal synchrony of body-related visual–tactile information in the absence of motor signals (e.g., by matching visual and tactile sensations arising at the same time related to their body), by their looking longer at synchronous, multisensory stimulation (Filippetti, Johnson, Lloyd-Fox, Dragovic, & Farroni, 2013). However, it remains unknown how sensitive newborns are to the spatial component of body-related information (e.g., can they map tactile and visual sensations referring to a specific part of their body). Research on older infants has highlighted the important role of spatial orientation and directionality for self–other discrimination from at least 3 months of age (Rochat & Morgan, 1995; Schmuckler, 1996), by showing discrimination of congruent and incongruent direction of leg movements (Rochat & Morgan, 1995) and detection of differences in canonical left/right directionality (Schmuckler, 1996). Despite the relevance of this previous infant research, which focused on various aspects involved in contingency detection in the first months of life, the basic mechanisms that allow for the construction and continuous update of one’s body representation throughout the lifespan are still unknown.

To address these issues, this study explored the looking behavior of human newborns in response to both congruent and incongruent spatial tactile cues with respect to corresponding visual feedback (see Figure1). We hypothesized that newborns would show a visual preference for the spatially congruent visual–tactile condition as compared to the incongruent condition, supporting our previous conclusions (Filippetti et al., 2013). Evidence of the detection of topographically specific body-related multisensory spatial integration from birth would represent a significant step toward a better understanding of processes involved in own-body perception from earliest stages of postnatal development. Furthermore, by exploring the specificity of visual–tactile detection, we aimed to provide for the first time a unified paradigm, which could allow comparisons across ages and represent a framework for future research on body perception in infancy and adulthood.

**Figure 1:**
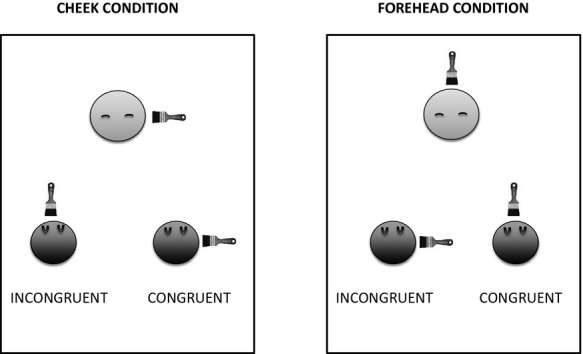
An example of the experimental paradigm used. Newborns were randomly assigned to either the “cheek” or the “forehead” conditions. In both conditions, visual–tactile congruent information was compared to visual–tactile incongruent information and multisensory stimulation was always temporally synchronous. In the congruent condition, the newborn was touched on the face (cheek or forehead) with a paintbrush on the specular congruent location with regard to the infant’s dynamic face displayed on the screen. In the incongruent condition, the newborn was again touched on the face (cheek or forehead), but the tactile stimulus was delivered on the incongruent location with regard to the brush stroke seen on the screen. Therefore, if the infant’s face on the video was touched on the forehead, the newborn’s cheek was touched simultaneously, and vice versa.

## Method

### Participants

The study was conducted at the Pediatric Unit of the Hospital of Monfalcone, Gorizia. Twenty newborns (8 female) from 12 to 94 h of age at time of test took part in the study; an additional 5 newborns also participated but were excluded due to fussiness (*n* = 2) or sleepiness (*n* = 3). All the newborns that completed the study met the screening criteria of normal delivery, had a birth weight >2,500 g, and had an Apgar score of at least 8 at 5 min. No abnormalities were present at birth.

The 20 newborns that participated in the experiment had a mean age of 40.45 h (*SD* = 2.39) at testing, with a mean gestational age of 40.14 weeks (*SD* = 0.90). The testing took place when the baby was awake and alert, usually during the hour preceding the feeding time. Parents were informed about the procedure and gave their consent to their child’s participation. The local ethics committee approved the study protocol.

### Materials

The newborns sat on the experimenters’ lap in a research room within the hospital. The distance between the monitor (69 cm) and the newborn’s head was approximately 30 cm. The newborn’s eye level was aligned to the center of the screen, and their eye movements were monitored through a video camera located on the top of the screen.

The visual stimuli presented consisted of two sets of identical previously recorded videos, one displayed on the left and other on the right of the screen. Identical paired videos were used to ensure newborns’ attention was fully engaged and to avoid sticky fixation. The videos contained an infant face being stroked with a paintbrush every 10 sec from a specular point of view, just as the neonate watching the screen would see her face being touched in front of a mirror (see Apps, Tajadura-Jiménez, Sereno, Blanke, & Tsakiris, 2015; Filippetti et al., 2013). Therefore, the viewing newborn’s side of the stroke always corresponded with the side of the face seen stroked on the screen (e.g., left infant’s cheek seen touched/right newborn’s cheek touched). Each stroke lasted approximately 1 sec. The experimental manipulation consisted of the location on the newborn’s face being touched (forehead versus cheek) with respect to the location on the face being touched in the video display. Hence, the newborns tested could be presented with either an infant’s face being stroked on the cheek (“cheek” condition) or on the forehead (“forehead” condition), while their corresponding cheek or forehead was stroked at the same time. Temporal synchrony was always held constant.

In order to measure sensitivity to spatial congruency, two videos were recorded, corresponding to the “cheek” and “forehead” conditions. In both the “cheek” and “forehead” conditions, the two identical faces subtended a visual angle of 17.2° × 18.1° each, and taken together, they subtended a visual angle of 17.2° × 58.4°. In all the stimuli, the pupil was 1 cm in diameter and the pairs of faces were 15 cm apart. The stimuli were presented using E-Prime 2.0.10. We chose a prerecorded unfamiliar infant face based on the assumption that newborns from 12 to 94 h have no experience of their own facial appearance (Filippetti et al., 2013).

Each newborn was presented with both the spatially congruent and incongruent conditions in a sequential looking procedure. Half of the sample (*n* = 10) was presented with the video of an infant’s face being stroked on the forehead, whereas the remaining half of the sample (*n* = 10) was presented with a video of an infant’s face being touched on the cheek. In the congruent condition, the newborn was touched either on the cheek or on the forehead with a paintbrush, and the strokes perfectly matched (in terms of temporal synchrony, location, and direction of movement) the brush stroke on the infant’s corresponding cheek or forehead displayed on the screen. In the incongruent condition, the newborn was again touched on the cheek or on the forehead, but the part of the infant’s face touched on the screen was manipulated, whereby if the newborn was touched on the cheek, the infant’s face on the screen was stroked on the forehead and vice versa. In the “cheek” manipulation, the stroke started on the middle of cheek and ended at the beginning of the ear. In the “forehead” manipulation, the stroke moved upward, started approximately 0.5 cm above the eyebrow line and ended at the edge of the forehead, in correspondence with the hairline.

### Procedure

The experiment began as soon as the newborn was seated on an experimenters lap and fixated upon the center of the screen. Each newborn was presented with one congruent and one incongruent trial, following a sequential procedure. The order of presentation of the two trials and the side of the stroke in the “cheek” condition (left/right cheek) were counterbalanced across infants. An experimenter delivered stroking manually standing behind the infant and out of view. The experimental procedure applied in the “forehead” and “cheek” conditions was identical unless otherwise specified. Each trial lasted 90 sec, interleaved with a 10-sec rest period (blank screen). During the rest period, a flashing icon was presented to maintain the newborn’s attention.

Each trial was divided into eight segments (approximately 10 sec each), where a segment started from the first frame that the paintbrush touched the infant cheek or forehead to the first frame in which the next paintbrush appeared on the screen. The video display presented the first stroke after approximately 3 sec of visual stimulus presentation, followed by 10 sec of video before the next stroke was presented, and so on. After the last stroke, approximately 6 sec of stimulus presentation followed. Thus, within a 90-sec trial, there were 8 strokes in total.

### Analyses

Based on the video recordings, two independent observers coded how long each newborn looked at the monitor (the first observer was naïve to the hypothesis) and total looking time was measured for each trial. The analysis was conducted by comparing the looking time values of the two coders. The coders were trained to code newborns’ looking behavior in the context of a different study and subsequently proceeded to an offline frame-by-frame coding of the present data, whereby looking time was analyzed by coding milliseconds of “engagement” and “disengagement” from the time code on the video display. Intraclass correlation coefficient (ICC) was performed on 20% of the sample and showed an agreement between coders = 0.93. Pearson’s r was 0.92.

Following Filippetti et al. (2013), we applied two exclusion criteria to the looking behavior. First, we took into account the effective interest of the infant by applying an offline infant-control procedure, and therefore, when the infant looked away for over 10 sec, the remaining section was discarded from the looking time analysis. Furthermore, we excluded the 10-sec segment if the newborn did not see the brush touching the infant face (“brush” procedure). As a consequence, if the newborn did not see the brush touching the infant’s face, the 10-sec segment that included this brush stroke was excluded from further analyses (Filippetti et al., 2013). Note that the final results remain the very similar if looking time during these segments is also included in the analyses (for the offline infant-control procedure, *t*(1,18)* *= 2.63, *p *=* *0.02; for the brush procedure, *t*(1,18) = 3.41, *p *=* *0.003; see Table1).

**Table 1:** Number of Trials Whose Data were Discarded Using the “Offline Infant-Control” Procedure and the “Brush” Procedure

Congruent condition			Incongruent condition		
Total trials	Excluded trials		Total trials	Excluded trials	
*N*	Infant-control	Brush	*N*	Infant-control	Brush
160	11	8	160	25	12

## Results

The looking time data were analyzed using percentage of looking time within each 90-sec trial. The results revealed greater looking time to the congruent condition (*M* = 0.47 sec, *SD* = 0.18) compared to the incongruent condition (*M* = 0.35, *SD* = 0.14), *(t*(1,18) =* 2.75, p *=* *0.01, *d* = 0.70). No effects of order of presentation (*F*(1,18) = 1.63, *p *=* *0.21) or location of the touch (cheek or forehead) (*F*(1,18) = 0.07, *p *=* *0.79) were observed.

## Discussion

Studies of body awareness in adults have provided compelling evidence for the role of multisensory integration in the construction of representations of one’s own body. Through the use of conflicting multisensory information, such as that which occurs in the rubber hand illusion (RHI) (Botvinick & Cohen, 1998) and in the enfacement illusion^1^ (Tsakiris, 2008), it has been proposed that both the temporal and the spatial properties of multisensory integration play a major role in updating and maintaining the mental representation of one’s own body (Longo & Haggard, 2012). Studies of infants’ abilities to detect the intermodal properties of body-related information have further demonstrated that multisensory integration is also involved in the early perceptual experience of the body (Bahrick & Watson, 1985; Morgan & Rochat, 1997; Reddy, Chisholm, Forrester, Conforti, & Maniatopoulou, 2007; Rochat & Morgan, 1995; Rochat & Striano, 2000; Schmuckler, 1996; Schmuckler & Jewell, 2007; Zmyj, Hauf, & Striano, 2009; Zmyj, Jank, Schütz-Bosbach, & Daum, 2011). In the present research, we corroborate and extend our previous findings (Filippetti et al., 2013) by demonstrating that not only temporal properties of multisensory information, but also spatial factors, are involved in the detection of body-related cues from birth.

Our findings with newborns are in accord with previous research in adults, in which it has been shown that the illusion of owning a specific body part only occurs when the fake limb matches the postural position of the real one (Tsakiris & Haggard, 2005). Recently, an fMRI adaptation of the enfacement illusion has shown that synchronous congruent visual–tactile information produces a stronger response to the illusion, compared to when the multisensory information is incongruent (Apps et al., 2015). Specifically, in this study, participants experienced tactile stimulation on their face, while they watched another face where tactile cues were delivered either synchronously or asynchronously, and at either the same skin location or at an incongruent location. Participants’ self-reports showed that the illusion of experiencing the other’s face as belonging to oneself was stronger in the synchronous and spatially congruent condition, compared to all of the other conditions (Apps et al., 2015). Therefore, the body surface sensory matching between seen and felt events provides crucial information for updating our body mental representation (Tsakiris, Costantini, & Haggard, 2008). Results of the present study provide the first evidence that detection of spatial congruent body-related information is present shortly after birth.

To our knowledge, this is the first study that specifically investigated sensitivity to spatial determinants in infancy in the absence of self-performed actions. Previous research has demonstrated infants’ discrimination of spatial congruency based on the matching between self-generated movements with its contingent visual feedback (Morgan & Rochat, 1997; Schmuckler, 1996). In the present study, using a previously recorded video of another infant’s face, we are able to exclude any possible influence from the contingent visual feedback of the newborns’ own self-generated actions, and thus, we provide evidence for the importance of spatial congruency prior to significant postnatal experience. Crucially, because the video did not mirror the newborns’ own movement, we demonstrate that even in the context of noncontingent visual-proprioceptive correspondence, newborns detect visual–tactile information related to the body, showing a preference for spatial congruent stimulation.

Although we cannot know whether or not the newborns in our study attributed the face in the video as their own, the present result provides valuable insights into our understanding of the precursors of body perception, as based on the adult literature. It could be argued that the multisensory visuo-tactile events (1 sec) were relatively rare as compared to the overall trial length (10 sec), thus introducing the possibility that newborns’ preferential looking was influenced by other features of the visual stimulus. Our assumption is that the multisensory visuo-tactile events during each trial were the most salient components and, therefore, most likely to influence their overall looking behavior. Because the ongoing visual input in between these salient events were identical for both groups, any differences in looking that we observed occurred despite the comparative rarity of the multisensory events.

It could also be argued that the preferential looking registered may be based on a unimodal visual–visual matching rather than a cross-modal visual–tactile integration. However, while we cannot completely exclude the possibility of an identification based on visual recognition during the “cheek” condition (based on newborns’ peripheral vision), we do not believe that the brush applied on the forehead could be seen by the infant. In fact, our exclusion criteria implied the rejection of segments where the newborn did not see the paintbrush on the screen, meaning that any looking behavior directed toward the live action was likely to be excluded from further analysis.

The present work, in line with Filippetti et al. (2013), shows a visual preference for multisensory integration, as opposed to previous infant research that demonstrated greater looking time for the noncontingent stimulation (e.g., Bahrick & Watson, 1985; Morgan & Rochat, 1997; Rochat & Morgan, 1995). Importantly, while these previous studies investigated the correspondence between visual and motor signals, in our studies, we have explored visual–tactile afferent information alone. We speculate that the direction of visual preference we observed can be explained by the tactile experience. Touch drives attention to the bodily self, while this does not necessarily happen when correspondence between visual and motor signals is experienced (see Filippetti, Lloyd-Fox, Longo, Farroni, & Johnson, 2014 for an analysis of cortical activation in response to these sensory stimuli in infants). We speculate that the presence of an initial focus on self-specifying information would help the infant to construct a reliable representation of the bodily self.

Future studies should examine the combined role of temporal and spatial properties of multisensory information for body perception at birth. In a recent study, Zmyj and colleagues have demonstrated the ability to combine temporal synchronous and spatial congruent body-related information at 10, but not at 7 months of age (Zmyj et al., 2011). In future research, it will be important to combine temporal synchrony and spatial congruency in order to address the origins of the interaction between these two components for body perception.

In conclusion, our study shows that spatial congruency between visual and tactile stimulation can be detected in newborns. Based on this and previous results (Filippetti et al., 2013), we propose that these basic abilities underpin the infant’s early perception of its own body and allow for the construction and continuous updating of body representations throughout the lifespan.

## Footnotes

1 The enfacement illusion refers to the illusory perception that another person’s face belongs to ourselves, as a consequence of repeated visual–tactile synchronous and congruent stimulation between the own and other faces (Tsakiris, 2008).
